# American Medical Association–Prize Essays for 1868

**Published:** 1867-12

**Authors:** 


### American Medical Association--Prize Essays for 1868.

The American Medical Association offers two prizes of *One Hundred Dollars* each, for the best two original essays upon subjects of professional interest; the Committee reserving the right to reject all unless deemed fully worthy.

Competitors for these prizes must forward their essays to Dr. Charles Woodward, Cincinnati, Ohio, free of expense, on or before the 1st of April, 1868.

Each essay must be accompanied by a sealed note containing the author's name and address, and on this sealed packet must be inscribed some sentiment, motto or device, corresponding to a like sentimeut, motto or device on the essay.

CHARLES WOODWARD, Chairman,

W. W. DAWSON,

E. B. STEVENS,

ROBERTS BARTHOLOW,

P. S. CONNOR,

} *Committee.*

